# Development of fermented and flavoured kefir milk

**DOI:** 10.1186/1753-6561-8-S4-P15

**Published:** 2014-10-01

**Authors:** Aline Melo, Maria Aparecida Silva

**Affiliations:** 1 Biotechnology - University of Sorocaba, Sorocaba, SP, Brazil; 2 Nutrition - University of Sorocaba, Sorocaba, SP, Brazil

## 

Kefir is a dairy beverage, obtained from lactic acid and alcoholic fermentation of its grains, which resembles yogurt in its flavour, aroma and consistency. It is composed of approximately 15-16 lactobacilli, approximately 7-9 streptococci/lactococci, 8 yeasts and 2 acetic bacteria (acetobacter). The fermenting action of Kefir bacteria and yeasts increases the biological value of milk, increasing synthesis of the B group vitamins, which help in the digestive process. The abundance in Calcium, Phosphor, and Magnesium is another of Kefir's characteristics, as for all other dairy products. It is an easily digested product that eliminates harmful bacteria and yeasts from the intestine, increases beneficial and protective bacterial population, and as a probiotic food has many therapeutic applications. This study had as its objective the production of fermented milk based on Kefir, when different flavours were tested, to have it sensorial tested to find the one with the highest acceptance. Firstly, an analysis of the total coliform content was performed, followed by a microbiological quantification analysis of the colony-forming-unit per gram (CFU/g), to ensure the quality of the product to be offered to tasters. Secondly, the Kefir milk was flavoured with 4 different flavours (passion fruit, strawberry, grape and mango), where juice from concentrate pulp and fruit bits were used, served to 60 tasters (students, professors and staff from UNISO), according to the model for rank-preference test (164/IV 'Testes Efetivos - Testes de preferência' Effective Test - PreferenceTests) according to the Instituto Adolfo Lutz.
